# Economic Stability of Children, a Key Social Determinant of Health, in the United States: Differences by Rurality

**DOI:** 10.1111/jrh.70164

**Published:** 2026-05-12

**Authors:** Elizabeth Crouch, Emma Boswell, Cassie Odahowski, Gabriel Benavidez, Kevin Bennett, Peiyin Hung, Joni Nelson

**Affiliations:** ^1^ University of South Carolina Rural Health Research Center Columbia South Carolina USA; ^2^ Department of Health Services Policy & Management University of South Carolina Columbia South Carolina USA; ^3^ Department of Public Health Baylor University Waco Texas USA; ^4^ Department of Family and Preventive Medicine University of South Carolina School of Medicine Columbia South Carolina USA; ^5^ Department of Stomatology, Division of Population Oral Health Medical University of South Carolina Charleston South Carolina USA

**Keywords:** economic stability, food security, health, housing, social determinants of health

## Abstract

**Purpose:**

This study aims to examine rural–urban differences in the prevalence of three measures of one domain of social determinants of heath, economic security, in a national sample of children.

**Methods:**

This was a cross‐sectional study (2022–2023) of the National Survey of Children's Health. Primary exposures included rurality and child and caregiver characteristics. Three economic stability outcomes were whether a child experienced housing instability, food insecurity, and/or income inadequacy (having a hard time getting by on family income). We used bivariate analyses and multivariable regressions analyses to examine the association between rurality and measures of economic stability. All analyses were weighted with survey sampling to generate nationally representative estimates.

**Findings:**

In the weighted multivariable regression analysis, adjusting for child and caregiver characteristics, rural children had higher odds of housing instability (aOR 1.20; 95% CI 1.07–1.34), food insecurity (aOR 1.47; 95% CI 1.22–1.78), and income inadequacy (aOR 1.32; 95% CI 1.18–1.48), compared to urban children.

**Conclusions:**

Rural children and their families are experiencing everyday challenges in housing, food access, and the ability to get by on their income, which are all shown to have ramifications on their health. Child advocates, policymakers, and program developers must consider these factors when developing programs and policies for families residing in rural America.

## 1 Introduction

Rural Healthy People 2030, a survey conducted in 2021–2022 of rural health stakeholders to identify the 20 Healthy People priorities that are most relevant for rural America, identified economic stability in the top 10 of the highest priorities for rural America [1, 2]. Economic stability, a key social determinant of health, refers to the ability of households to afford foods that are nutritious, utilize healthcare, and afford stable housing [2]. More recently, economic hardship has been understood as an adverse childhood experience and is associated with poor physical and mental health into adulthood [3].

More than 20% of rural children live in poverty, and around 12% of rural children live in deep poverty [4]. Prior research has found that rural children residing in high poverty are less likely to utilize healthcare than their urban counterparts [5]. Housing instability, one measure of economic instability, has been associated with several poor health outcomes, including a higher likelihood of mortality and lower rates of good physical and mental health [6]. Children experiencing housing instability are less likely to receive both necessary and preventive medical care [7]. Children experiencing housing instability also have a higher likelihood of experiencing food insecurity [8].

Housing instability is not distributed equally among groups—eviction rates are higher in rural counties, in households where the rent is a higher proportion of their income, and in households with children [9]. Rural residents are also more likely to have lower quality housing, coupled with increasing cost, particularly in high‐amenity areas where seasonal residents drive up prices [10]. This amenity trap means that rural residents who are service workers in that area may be priced out of their communities.

Limited research has examined the relationship between rurality and specific, everyday measures of economic stability of children, such as housing instability, food insecurity, and how hard it is to get by on income. Understanding these structural factors is key for resource allocation in policies and programs. This information would be especially helpful for social workers, teachers, school counselors, and other child advocates to see what rural children are facing, compared to their urban counterparts. Therefore, the purpose of this study is to examine the prevalence of three measures of economic stability—housing instability, food insecurity, and income inadequacy—in a national sample of children, and to evaluate whether this differs between rural and urban residents, and to examine the associations between rurality and measures of economic security. The findings from this study will be useful in describing the need for programs and policies to improve economic stability of rural families and their children in the United States.

## 2 Methods

This study uses cross‐sectional data from the 2022–2023 National Survey of Children's Health (NSCH), which is a population‐based survey administered by the US Census Bureau across all 50 states (https://www.childhealthdata.org/). Caregivers are asked about one child in their household ages 0 to 17 years of age, with questions encompassing information about their child, their household situation, and a variety of other areas. The NSCH oversamples children under 5 years of age and children with special healthcare needs. For the 2022–2023 dataset, 109,625 children were included in the sample. The NSCH reports all data in terms of the child, as this is the population around which the survey is designed.

Utilizing the Strengthening the Reporting of Observational Studies in Epidemiology (STROBE) reporting guidelines, this study used publicly available data without any identifying characteristics of the respondents. Therefore, this study did not include human subjects research and was deemed exempt from the institutional review board of the University of South Carolina.

The NSCH includes four geographic variables: state of residence, core‐based statistical area status, metropolitan statistical area status, and metropolitan principal city status. For states with small sample sizes, core‐based statistical area status, metropolitan area status, and metropolitan principal city status are suppressed; therefore, residence information is missing for 20,452 children. Using these geographic variables, four levels of rurality were constructed: noncore rural (no town with 10,000 or more persons), micropolitan rural (a town with more than 10,000 but fewer than 50,000 residents), urban (urbanized population of 50,000 people or more but does not include the principal county in a metropolitan area), and urban core (the principal county in a metropolitan area); these were then dichotomized into rural and urban counties per the Office of Management and Budget [11]. The final sample size included 88,813 children.

Measures of economic stability included housing instability, food insecurity, and whether it was hard to get by on family income [12]. Housing instability was a composite measure, where a child was categorized as having experienced housing instability if their caregiver responded affirmatively to any of the following: that they ever experienced homelessness or lived in a shelter, if their caregiver missed a mortgage payment in the last year, or if they have lived in three or more places in the last 12 months. Food insecurity was dichotomized into two groups: whether a child could always afford to eat (including whether that meal was a good nutritious meal or not always the kinds of food they should eat) or whether a child sometimes or often could not afford enough to eat. Hard to get by on family income was grouped by the following responses: (1) never or rarely hard to get by on family income or (2) somewhat or very often hard to get by on income.

Potential confounding variables were selected using Andersen's Behavioral Model of Health Care Utilization [13] and included the following: child gender, age, race/ethnicity, and whether the child had special healthcare needs, using the NSCH special healthcare needs indicator. Additional confounding variables included primary language spoken in the home, caregiver education, family structure, federal poverty level, and insurance type. Descriptive statistics and bivariate analyses were used in the estimation of column frequencies, percentages, and associations, with an alpha value of 0.05. Multivariable regression models were used to predict the following outcomes: living in a household with housing instability, food insecurity, and hard to get by on family income. Federal poverty level and type of health insurance were not included in the regression models, due to potential high collinearity with the outcome measures of housing instability, food insecurity, and hard to get by on income. All survey sampling methods instructed in the NSCH were performed, including sampling weight, strata, and cluster. Analyses were performed using SAS v9.4 (SAS Institute Inc., Cary, NC, USA).

## 3 Results

The study sample was mostly male (51.2%), while over a third of the sample of children were 5 years old or less (39.2%), and non‐Hispanic White ethnicity/race (46.9%, Table 1). One‐fifth (20.6%) of the children had special healthcare needs and 15.7% of the children did not speak English as the primary language in their home. Most children lived in a household where the caregiver had some college education or more (72.4%) and lived with two parents, currently married (62.8%). Less than 20% of the children lived below the federal poverty level (18.6%). Note that 29.1% of the children had public health insurance.

**TABLE 1:** Characteristics of respondents to the 2022–2023 National Survey of Children's Health, in total and stratified by rurality (*n* = 88,813).

Characteristic	All (%)	Rural (%)	Urban (%)	*p* value
		50.9	29.6	
Characteristics of child				
Sex of child				0.05
Male	51.2	51.3	51.1	
Female	48.3	48.7	48.9	
Age of child (years)				0.35
0–5	39.2	38.1	39.3	
6–12	30.0	30.6	30.0	
13–17	30.8	31.3	30.7	
Race/ethnicity of child				**<0.0001**
Non‐Hispanic White	46.9	68.4	44.3	
Non‐Hispanic Black	12.8	9.2	13.2	
Hispanic	27.6	14.8	29.1	
Non‐Hispanic Other	12.8	7.5	13.4	
Special healthcare needs, yes	20.6	21.7	20.5	0.09
Characteristics of the caregiver				
Primary language				**<0.0001**
Not English	15.7	7.7	16.7	
Caregiver education				**<0.0001**
Less than high school or high school	27.6	37.2	26.4	
Some college or more	72.4	62.8	73.6	
Family structure				**<0.0001**
Two parents, currently married	62.8	59.5	63.2	
Two parents, not currently married	6.7	7.5	6.6	
Single parent	22.7	22.9	22.7	
Other	7.8	10.1	7.5	
Federal poverty level (%)				**<0.0001**
0–99	18.6	23.7	18.0	
100–199	19.7	26.2	18.8	
200–399	28.7	31.8	28.3	
>400	33.0	18.3	34.8	
Insurance				**<0.0001**
Public health insurance only	29.1	39.8	27.8	
Private health insurance only	56.6	42.9	58.3	
Public and private insurance	5.2	6.7	5.0	
Uninsured	9.1	10.5	8.9	

*Note*: Bold values indicate statistical significance at *p* < 0.05, calculated from Pearson's chi‐square tests.

A lower percentage of rural children, compared to urban children, were non‐Hispanic Black (9.2% vs. 13.2%), Hispanic (14.8% vs. 29.1%), or non‐Hispanic other (7.5% vs. 13.4%, *p* < 0.0001). A lower percentage of the rural children did not speak English as their primary language in their home, than urban children (7.7% vs. 16.7%, *p* < 0.0001). A smaller proportion of the rural children resided in a home with two parents, currently married (59.5%) than urban children (63.2%, *p* < 0.0001). Rural children were more likely to live below the federal poverty level than their urban counterparts (23.7% vs. 18.0%, *p* < 0.0001), as well as have public health insurance (39.8% vs. 27.8%, *p* < 0.0001).

A little more than 17.3% of the children experienced housing instability, and just 5.3% of the children sometimes or often could not afford enough to eat (Table 2). Just over 14% of the children somewhat or very often had caregivers reporting that they found their family income hard to get by on. Compared to urban children, rural children experienced all three measures of economic stability at a higher rate: housing instability (19.4% vs. 17.0%, *p* = 0.0035), food insecurity (7.1% vs. 5.1%, *p* = 0.001), and somewhat or very often hard to get by on family income (17.9% vs. 13.2%, *p* < 0.0001).

**TABLE 2:** Housing instability, food insecurity, and hard to get by on family income, as reported by respondents to the 2022–2023 National Survey of Children's Health, in total and stratified by rurality (*n* = 88,813).

Characteristic	All	Rural (%)	Urban (%)	*p* value
Housing instability				0.0035
Experienced housing instability	17.3	19.4	17.0	
Did not experience housing instability	89.0	80.6	83.0	
Food insecurity				0.0001
Could always afford to eat	94.7	92.9	94.9	
Sometimes or often could not afford enough to eat	5.3	7.1	5.1	
Hard to get by on family income				<0.0001
Never or rarely	85.7	82.1	86.2	
Somewhat or very often	14.3	17.9	13.2	

*Note*: *p* values were calculated from Pearson's chi‐square tests, and *p* < 0.01 was deemed statistically significant.

In adjusted multivariable regression analysis, rural children had higher odds of housing instability than urban children (aOR 1.20; 95% CI 1.07–1.34, Table 3). Rural children had greater odds of food insecurity (aOR 1.47; 95% CI 1.22–1.78) compared to urban children. Furthermore, rural children had higher odds of having a hard time getting by on family income (aOR 1.32; 95% CI 1.18–1.48).

**TABLE 3:** Prediction of housing instability, food insecurity, hard to get by on family income, and not receiving preventive care by respondents to the 2022–2023 National Survey of Children's Health (*n* = 88,813).

Characteristics	Predicting housing instability		Predicting food insecurity		Predicting hard to get by on family income	
Rurality	PE	95% CI^a^	PE	95% CI^a^	PE	95% CI^a^
Rural	**1.20**	**1.07−1.34**	**1.47**	**1.22−1.78**	**1.32**	**1.18−1.48**
Urban	(Referent)	(Referent)	(Referent)	(Referent)	(Referent)	(Referent)
Child characteristics						
Race/ethnicity						
White, non‐Hispanic	(Referent)	(Referent)	(Referent)	(Referent)	(Referent)	(Referent)
Black, non‐Hispanic	**2.21**	**1.97−2.47**	**1.87**	**1.52−2.31**	**1.35**	**1.18−1.54**
Hispanic	**1.79**	**1.61−2.00**	**1.83**	**1.51−2.21**	**1.41**	**1.25−1.58**
Other, non‐Hispanic	1.09	0.98−1.22	1.18	0.96−1.45	0.95	0.84−1.07
Child sex						
Male	(Referent)	(Referent)	(Referent)	(Referent)	(Referent)	(Referent)
Female	1.02	0.95−1.10	**1.19**	**1.03−1.37**	1.00	0.92−1.09
Child age (years)						
0–5	(Referent)	(Referent)	(Referent)	(Referent)	(Referent)	(Referent)
6–11	0.91	0.83‐1.00	0.84	0.71‐1.00	0.96	0.86‐1.06
12–17	**2.66**	**2.46−2.88**	0.79	0.66−1.00	1.11	1.00−1.23
Special healthcare needs						
Yes	**1.59**	**1.46−1.73**	**1.73**	**1.49−2.02**	**1.84**	**1.68−2.02**
No	(Referent)	(Referent)	(Referent)	(Referent)	(Referent)	(Referent)
Primary language						
English	**0.75**	**0.65−0.86**	0.96	0.76−1.22	0.89	0.77−1.03
Not English	(Referent)	(Referent)	(Referent)	(Referent)	(Referent)	(Referent)
Characteristics of caregiver/household						
Guardian education						
High school diploma or less	**1.59**	**1.44−1.75**	**1.86**	**1.58−2.19**	**1.76**	**1.59−1.95**
Some college or more	(Referent)	(Referent)	(Referent)	(Referent)	(Referent)	(Referent)
Family structure						
Two parents, currently married	(Referent)	(Referent)	(Referent)	(Referent)	(Referent)	(Referent)
Two parents, not currently married	**2.49**	**2.17−2.87**	**2.72**	**2.14−3.47**	**2.22**	**1.91−2.58**
Single parent	**2.33**	**2.12−2.57**	**2.61**	**2.14−3.19**	**2.52**	**2.66−2.80**
Other	**2.08**	**1.78−2.44**	**2.12**	**1.58−2.84**	**1.64**	**1.39–1.94**

*Note*: Bold values indicate statistical significance at *p* < 0.05.

Abbreviation: PE, point estimate.

^a^ 95% CI = 95% Wald confidence intervals.

### 3.1 Other Factors Associated With Three Measures of Economic Instability

Non‐Hispanic Black children and Hispanic children had a higher likelihood of housing instability (aOR 2.21; 95% CI 1.97–2.47; aOR 1.79; 95% CI 1.61–2.00), food insecurity (aOR 1.87; 95% CI 1.52–2.31; aOR 1.83; 95% CI 1.51–2.21), and a hard time getting by on family income (aOR 1.35; 95% CI 1.18–1.54; aOR 1.41; 95% CI 1.28–1.58) than non‐Hispanic White children. Female children had greater odds of food insecurity than male children (aOR 1.19; 95% CI 1.03–1.07). Children 12–17 years of age had greater odds of housing instability than children 0–5 years of age (aOR 2.66; 95% CI 2.46–2.88).

Children with special healthcare needs were more likely to have housing instability (aOR 1.59; 95% CI 1.46–1.73), food insecurity (aOR 1.73; 95% CI 1.49–2.02), and a hard time getting by on family income (aOR 1.84; 95% CI 1.68; 95% 1.68–2.02) than children without special healthcare needs. Children who resided in a home where English was the primary languages were more likely to have housing stability (aOR 0.75; 95% CI 0.65–0.86) than children whose primary language was not English. Children whose caregiver had a high school diploma or less had greater odds of housing instability (aOR 1.59; 95% CI 1.44–1.75), food insecurity (aOR 1.86; 95% CI 1.58–2.19), and income inadequacy (aOR 1.76; 95% CI 1.59–1.95) than children whose caregiver had some college education or more. Every category of family structure had higher odds of housing instability, food insecurity, or income inadequacy than families with two parents who are currently married.

## 4 Discussion

This is the first study, to our knowledge, to examine the prevalence of economic stability, specifically housing instability, food insecurity, and income inadequacy, in a national sample of children, and whether this prevalence differs between rural and urban residents. These findings are important, as most social determinants of health research focus on one element of economic stability, rather than looking at multiple measures of economic stability simultaneously. While rural poverty has certainly been studied, studies examining concrete measures of rural poverty such as housing instability and food insecurity are dated, as earlier research had found that housing stock was more likely to be substandard in a rural area and that housing costs were more likely to take up a disproportionate share of household income in rural areas, compared to those residing in an urban area [14]. Thus, it is important to document the finding that higher rates of rural poverty contributed to all three outcomes—rural children are more likely to experience housing instability, food insecurity, and having a hard time getting by on their family income.

The most significant difference observed was income inadequacy—a child in a household having a hard time getting by on income. Excessive inflation in the post‐pandemic era, leading to rising costs of real estate and food disproportionate to income, only exacerbates these issues [15]. Furthermore, fewer resources, such as the supply of low‐income housing and landlords accepting housing vouches/subsidized housing, and fewer local food assistance programs and food pantries, create additional barriers with housing and food security for rural residents [16]. This only increases stress on low‐income families [17].

This information is vital to disseminate, as negative downstream effects of economic instability have been well documented. Economic instability has been shown to increase parental stress [18] and has been linked to negative health outcomes [3]. Furthermore, economic instability also creates competing priorities when accessing healthcare, which can delay obtaining important preventive services, including well‐child visits and dental prophylaxis [19]. Families facing economic instability must weigh the costs of necessities (such as housing and food) and obtaining such preventive oral healthcare [19]. Describing how poverty affects the day‐to‐day lives of families, through housing, food, and restricted income, helps to illuminate the need for policies and programs that can directly impact the financial well‐being of families, particularly those living in rural areas who, in many cases, also face transportation challenges to accessing assistance.

Policy changes are needed to combat rural economic stability and improve health outcomes for rural children and their caregivers. The National Rural Health Association has advocated that economic development be a key part of improving the health of rural Americans [20]. One example of an economic development strategy is the CARE (Creation, Retention, Attraction, and Expansion) model, which aims to assist entrepreneurs and small businesses in the community, as well as attracting new industry [21]. With hospitals being the second largest employer in many rural areas, government loans, expansion of government insurance such as Medicaid in non‐expansion states, and other measures to keep rural healthcare accessible is another solid economic development strategy [22]. Future policy research is needed to expand on how best to improve the health of rural children through economic development policies, and the findings from this study demonstrate the need for such additional research.

This study has a variety of strengths and weaknesses. This study is cross‐sectional; whether rural‐urban disparities in economic instability persist over time requires further investigation. The caregiver is the respondent for the child, so full information on and from the child may not be known. Social desirability bias may also come into play, as respondents may be uncomfortable sharing information about economic instability. Survey methods from the NSCH of sampling only uses address‐based samples, which means that families that are homeless or transient are not included in the survey, so the prevalence of housing instability will be underestimated, and the findings of this study are not generalizable to these groups. Finally, the NSCH suppresses some geographic information for respondents in states with a smaller population, so the sample size did decrease by over 20,000 children. This study also has several strengths, including innovation, as it is among the first to use the new NSCH variable on housing instability. To our knowledge, this is the first study to examine housing instability, by rurality, using a national sample of children.

## 5 Conclusions

The widespread prevalence of intersecting economic challenges facing rural children further supports the importance of economic stability as a central priority in *Rural Healthy People 2030*, not only for the overall development of rural communities but also for the health and well‐being of children. Rural children and their families face persistent challenges related to housing quality, food access, and meeting basic financial needs, each of which has well‐documented consequences for child health. Child advocates, policymakers, and program developers must account for these structural factors when designing economic development programs and policies to support families living in rural America.

## Conflicts of Interest

The authors declare no conflicts of interest.

## Funding

This study received no funding.
